# Supplementary material for: Age Estimation in Sepia officinalis Using Beaks and Statoliths
Source: Animals (Basel). 2024 Jul 31;14(15):2230. doi: 10.3390/ani14152230 (PMC11311034; doi:10.3390/ani14152230)
Supplement: Supplementary file 1 [file animals-14-02230-s001.zip › animals-3072430-supplementary.pdf]

Supplementary Material

Supplementary Table S1 Equations parameters and AIC value for each model tested for the relationship between number of increments (NI) counted in the upper beak versus dorsal mantle length (ML) and total weight (TW) of *Sepia officinalis*. Linear equation y=*a + bx;* Exponential equation y=*a**e^{bx}; Power equation y=*a*x^{b}; Logarithmic equation y=*a*ln(x)+*b*.

SEX	VARIABLE	MODEL	AGE			
			a	***B***	**AIC**	**R^2**
**Males**	**ML**	**Linear**	30.99	0.58	567.15	0.89
		**Exponential**	3.97	0.005	-84.34	0.83
		**Power**	1.09	0.73	**-114.33**	0.90
		**Logarithmic**	-286.17	82.18	583.18	0.86
	**TW**	**Linear**	-183.79	2.61	820.12	0.82
		**Exponential**	3.005	0.013	59.76	0.82
		**Power**	-4.61	1.95	**34.22**	0.87
		**Logarithmic**	-1462.3	338.5	863	0.68
**Females**	**ML**	**Linear**	26.22	0.633	668.12	0.91
		**Exponential**	3.95	0.005	-111.15	0.86
		**Power**	0.95	0.76	**-146.85**	0.91
		**Logarithmic**	-316.27	88.83	703.07	0.87
	**TW**	**Linear**	-287.94	3.64	1069.47	0.77
		**Exponential**	2.96	0.014	62.39	0.86
		**Power**	-5.20	2.097	**35.66**	0.90
		**Logarithmic**	-2049.8	468.2	1114.56	0.62

Supplementary Table S2 Equations parameters and AIC value for each model tested for the relationship between number of increments (NI) counted in statoliths versus dorsal mantle length (ML) and total weight (TW) of *Sepia officinalis*. Linear y=*a + bx;* Exponential y=*a**e^{bx}; power y=*a*x^{b}*;* logarithmic y=*a*ln(x)+*b*.

SEX	VARIABLE	MODEL	AGE			
			A	***b***	**AIC**	**R^2**
**Males**	**ML**	**Linear**	-2.55	0.83	316.68	0.86
		**Exponential**	3.61	0.007	**-66.77**	0.92
		**Power**	0.07	0.94	-58.08	0.90
		**Logarithmic**	-376.2	100.5	330.47	0.80
	**TW**	**Linear**	-270.74	3.35	472.49	0.62
		**Exponential**	2.04	0.02	**17.49**	0.90
		**Power**	-7.39	2.51	22.21	0.89
		**Logarithmic**	-1687.9	386.3	480.42	0.53
**Females**	**ML**	**Linear**	9.85	0.75	386.92	0.81
		**Exponential**	3.69	0.007	**-54.87**	0.90
		**Power**	0.52	0.85	-52.63	0.87
		**Logarithmic**	-316.9	88.79	395.30	0.77
	**TW**	**Linear**	-213.44	3.065	575.42	0.52
		**Exponential**	2.38	0.01	**45.63**	0.87
		**Power**	-5.861	2.22	45.72	0.83
		**Logarithmic**	-1474.4	346.8	580.67	0.46
